# Bioremediation of Heavy Metals by Rhizobacteria

**DOI:** 10.1007/s12010-022-04177-z

**Published:** 2022-10-26

**Authors:** Roohallah Saberi Riseh, Mozhgan Gholizadeh Vazvani, Najmeh Hajabdollahi, Vijay Kumar Thakur

**Affiliations:** 1 Department of Plant Protection, Faculty of Agriculture, Vali-E-Asr University of Rafsanjan, Imam Khomeini Square, Rafsanjan, 7718897111 Iran; 2 Biorefining and Advanced Materials Research Center, Scotland’s Rural College (SRUC), Edinburgh, EH9 3JG UK; 3 School of Engineering, University of Petroleum & Energy Studies (UPES), Dehradun, 248007 India; 4 Centre for Research and Development, Chandigarh University, Mohali, 140413 Punjab India; 5 Department of Biotechnology, Graphic Era Deemed to Be University, Dehradun, 248002 Uttarakhand India

**Keywords:** PGPRs, Negative interaction, Heavy metals, Bioremediation, Mediator

## Abstract

Heavy elements accumulate rapidly in the soil due to industrial activities and the industrial revolution, which significantly impact the morphology, physiology, and yield of crops. Heavy metal contamination will eventually affect the plant tolerance threshold and cause changes in the plant genome and genetic structure. Changes in the plant genome lead to changes in encoded proteins and protein sequences. Consuming these mutated products can seriously affect human and animal health. Bioremediation is a process that can be applied to reduce the adverse effects of heavy metals in the soil. In this regard, bioremediation using plant growth–promoting rhizobacteria (PGPRs) as beneficial living agents can help to neutralize the negative interaction between the plant and the heavy metals. PGPRs suppress the adverse effects of heavy metals and the negative interaction of plant-heavy elements by different mechanisms such as biological adsorption and entrapment of heavy elements in extracellular capsules, reduction of metal ion concentration, and formation of complexes with metal ions inside the cell.

## Introduction

The rhizosphere is an area with a strong chemical relationship between plant roots and soil microorganisms. The interaction between plant roots and soil microorganisms results in a synergistic relationship that can increase the yield and productivity of plants. This relationship can also help plants withstand various stresses [1]. Among the biotic and abiotic stresses that plants face during their growth stages can be pointed to pests, fungi, bacteria, viruses, nematodes, drought, salinity, flooding stress, and heavy metal pollution [2].

Heavy metals (HMs) or trace metals such as arsenic (As), aluminum (Al), cadmium (Cd), chromium (Cr), beryllium (Be), mercury (Hg), copper (Cu), lead (Pb), iron (Fe), nickel (Ni), zinc (Zn), and thallium (Tl) enter the environment through industrial activities and contaminate groundwater, disrupt the food chain, reduce food quality, and threaten human health. These HMs disrupt the operation of pivotal cellular components [3–6]. These elements have relatively high density and are toxic even at low concentrations [7]. The chemical pollutants are even released into the atmosphere by anthropogenic activities. After entering into the soil, most inorganic pollutants do not degrade by chemical or microbial agents, and stay stable for long periods [8].

Metal contamination destroys ecosystems and has adverse effects on human health through entering the food chain, groundwater aquifers, and touching and handling polluted soil. This contamination reduces production and food quality and reduces culturable and fertile lands for farming [7, 9, 10]. Soils can be polluted by HMs and other different ways like exposure to the high metal waste, pesticides, mine tailings, coal fuel residues, paints, leaded gasoline, manures, synthetic fertilizers, petrochemical spillage, wastewater irrigation, sewage sludge, and deposition of atmosphere. On the other hand, air pollution through greenhouse gas emissions is increasing rapidly [11, 12].

**Fig. 1:**
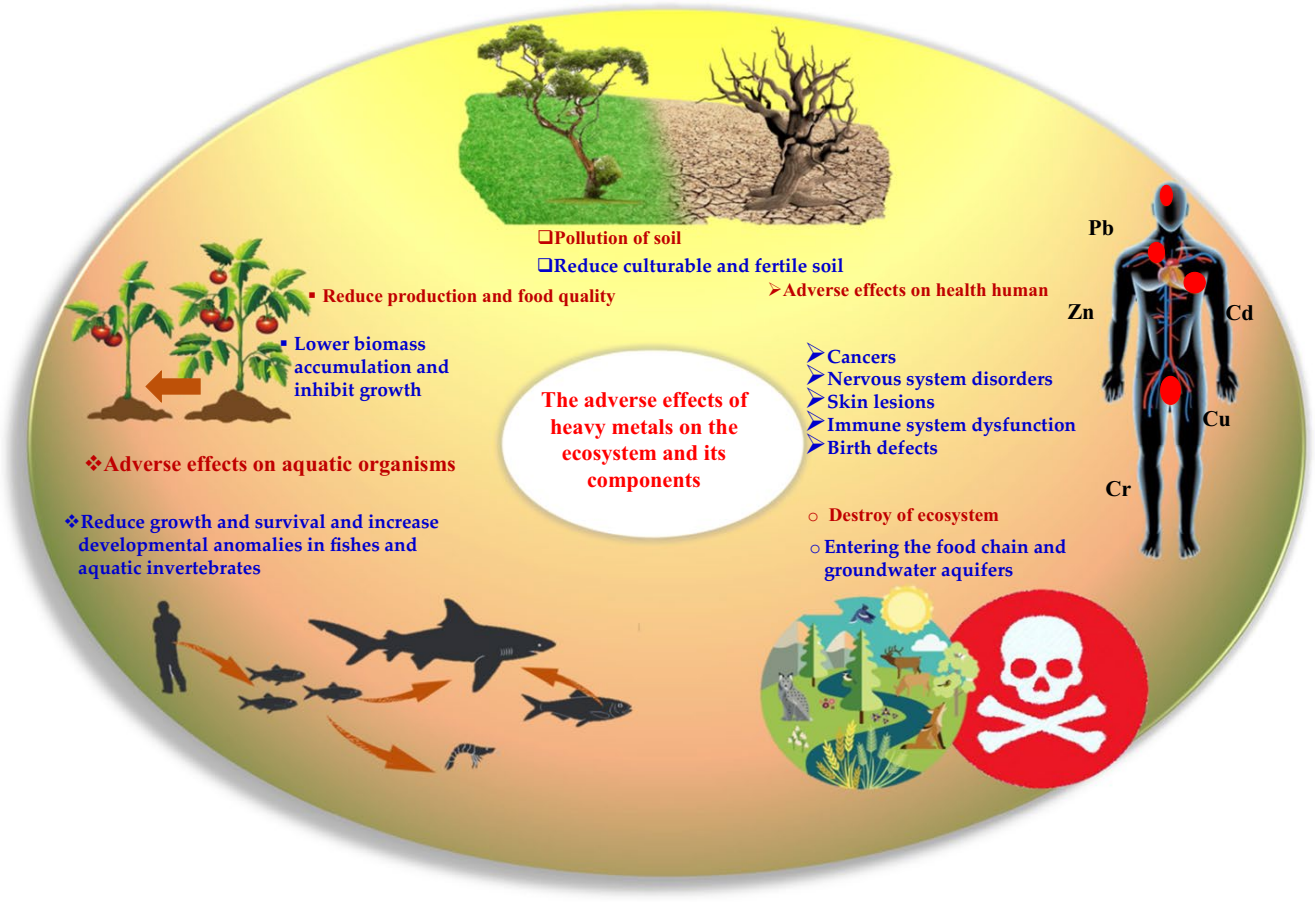
Different adverse effects of heavy metals on environmental components

When HMs enter the water, soil, and air, plants and aquatic organisms absorb them, and their physiological and functional activities can be affected. These metals in fishes and aquatic invertebrates reduce growth and survival and increase developmental anomalies. Also, in plants, these metals lower biomass accumulation, inhibit growth and photosynthesis, alter the balance of water, assimilate nutrients, and cause senescence, chlorosis, and plant death [13, 14]. Different effects of heavy elements on human health have been observed, such as nervous system disorders, skin lesions, immune system dysfunction, cancer, and birth defects [15]. Different adverse effects of HMs on environmental components are schematically depicted in Fig. 1.

Beneficial organisms in the rhizosphere increase the plant’s ability to eliminate the adverse effects of HMs [1]. There are many benefits to studying the rhizosphere, such as identifying beneficial microorganisms (as biocontrol agents) to suppress plant diseases and weeds. The abilities of *Pseudomonas fluorescens* (strains VUPF5, VUPF52), *Bacillus cereus* strain (PRC95), and *Bacillus subtilis* (strain PRC96) were tested as biocontrol agents for *Meloidogyne incognita* on the pistachio cultivars. Four months after nematode inoculation in seedlings of cultivars treated by bacterial strains, numbers of galls, egg masses, and second juveniles decreased compared with the non-treated seedlings [16]. This refers to the high survival of rhizosphere bacteria in the soil.

One commonly used biopolymer for biological control of plant pathogens is alginate. This biopolymer with biocompatibility, biodegradability, and long-term performance can be used in encapsulation of bacteria [17]. In a research, 2 strains of *Pseudomonas fluorescens* (VUPF5 and T17-4) were used in alginate–gelatin nanocomposite beads. The ability of these components was examined (after 60 days) in control of fusarium disease in potato in vitro and in vivo conditions. These strains reduced disease incidence [18]. Also, beneficial microorganisms in contaminated soils act as bioremediation agents by forming stable soil aggregates. Bacterial encapsulation in natural coatings (electrospun cyclodextrin fibers (CD-F), gellan gum microbeads, hydrogel-encapsulated, and carboxymethyl cellulose) has been used to bioremediate heavy metals [19–22].

Bioremediation by microbes or their enzymes transforms toxic HMs into more minor toxic forms and helps to clean up the contaminated environments [23]. Plasmids and bacterial chromosomes contain resistance genes against many HM cations [23–25]. Bacterial plasmids encode resistance systems for toxic metal ions such as Ag, Cd, Co, Cu, Hg, Ni, and Pb. The functional of these systems is based on the energy-dependent efflux of toxic ions [26]. Bioremediation by bacteria includes different mechanisms such as biosorption of HMs to the cell wall and trapping in extracellular capsules, precipitation, the flow of metal ions outside the cell, reduction of HM ions to a less toxic state accumulation, and metal ion complexation inside the cell, which resulted to the adsorption of HM ions [23, 27, 28].

**Fig. 2:**
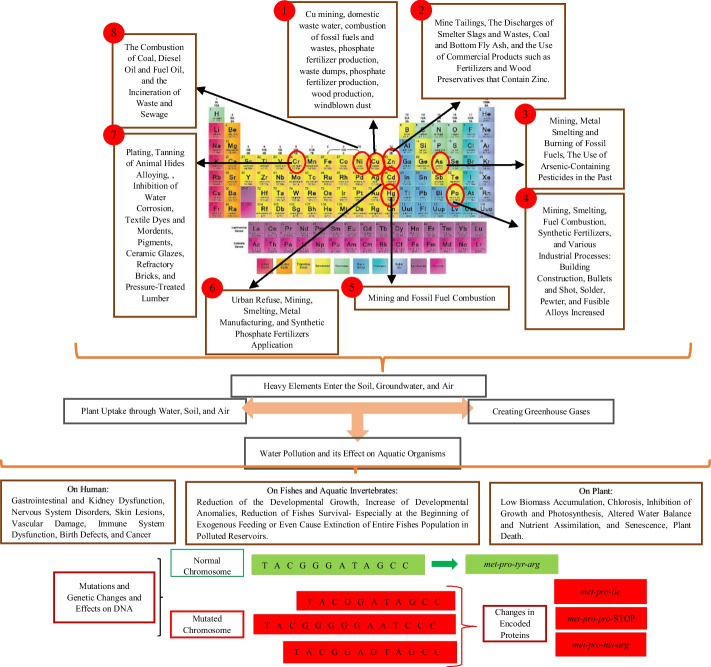
Heavy metal source and their effects on living organisms

Figure 2 shows the types of heavy metals in the periodic table and their applications in the industry. This figure shows the use of heavy metals in industries and how these metals affect the environment and what the consequences are.

In this review, we discuss HMs and their effect on plants. Also, we deal with the bioremediation of HMs by rhizobacteria and the mechanisms these bacteria use to remediate these toxic effects.

### Rhizosphere and Its Importance

The rhizosphere is rich in nutrients and has chemical and biological activities that is influenced by plant roots. Various macro and microorganisms (bacteria, viruses, fungi, protozoa, algae, nematodes, and microarthropods) co-exist in the rhizosphere and have different interactions between macro-microorganisms and the plant. The plant growth–promoting rhizobacteria affect the plant and its yield [29]. These rhizobacteria play a necessary role in plant functions in the rhizosphere by influencing physiology and development [30].

The rhizosphere is subdivided into three zones, including:

1. endorhizosphere which includes endodermis and cortical layers of the root;
2. rhizoplane, which is the root surface and soil particles, and microbes adhere to it (soil particles and microbes in the root surface, epidermis, cortex, and mucilaginous polysaccharide layer); and
3. and ectorhizosphere, which is the soil adjacent to the roots [29].

In fact, with the growth of roots and the presence of rhizobacteria, the external tissues and soil change physically, chemically, and biologically, that the result is the above three cases.

### Rhizosphere Beneficial Bacteria

Plant growth–promoting rhizobacteria (PGPRs) can promote plant growth and active defense systems in plants [31]. Plant–PGPR interaction is an important factor in determining plant health and soil fertility is in the rhizosphere. A group of rhizospheric bacteria is denominated as plant growth–promoting rhizobacteria because they affect plant growth and yield of commercially main crops. PGPRs contain different genera like *Arthrobacter*, *Azotobacter*, *Azospirillum*, *Burkholderia*, *Bacillus*, *Klebsiella*, *Enterobacter*, *Pseudomonas*, *Xanthomonas*, *Serratia*, and *Streptomyces* [25, 32, 33].

The roles of PGPRs can be summarized as follows:

1. Nutrient acquisition in plants
2. The growth of plants by producing and secretion of plant growth-promoting substances (auxins, gibberellins, and cytokinins)
3. Enhancing plant growth-promoting microbes
4. Production of phytohormones
5. Solubilization of nutrients (PO^4−^, Fe^2+^, and Fe.^3+^)
6. Metal toxicity reduction
7. Elicitating root metabolic activities through bacterial surface components, by biologically nitrogen fixation
8. Disease resistance interactions in plants [32, 34, 35]

Bacteria with their small size are the most numerous inhabitants in rhizosphere. In Gram-negative bacteria, the genus Pseudomonas is the most efficient root colonizer. In the per gram of rhizosphere, soil approximately consists of 10^8^–10^12^ bacterial cells [32].

The above features have made PGPRs as effective colonizers of plant roots, which can be applied in a variety of processes such as bio-fertilization, bioenergy production, and bioremediation [32]. Motility and chemotaxis play a key role in exerting PGPRs’ beneficial effects [34, 35].

### Environmental Contaminants

Environmental contaminants are chemicals that enter the environment due to human activities. Some of these contaminants do not decompose easily due to their high stability. After releasing these substances, they enter the food chain and disrupt this cycle. Natural chemicals are another group of environmental contaminants that industrial activities may increase their mobility and cause their entrance to the food chain at higher concentrations. Some environmental contaminants such as metals, ionic species (e.g., perchlorates), and organic matter (carbon-based) have been detected in foods and named persistent organic pollutants. The term “stable organic pollutants” is because these substances stay in the environment for an extended time without breaking down [36]. Different HMs have different effects on health depending on their level of contamination in food. In general, HMs in plants affect morphological, physiological, and reproductive properties. In humans, cadmium and mercury cause kidney damage, chronic toxicity, poor reproductive capacity, high blood pressure, tumors, and liver disorders. Lead also causes renal failure and liver damage. High concentrations of zinc and copper cause nephritis, anuria, and extensive lesions in the kidney [37].

### Contamination of Soil with Heavy Elements and Their Effect on Plants

Contamination of soil by HMs due to the increase in geologic, anthropogenic, and industrial activities may harm humans and other living organisms in the ecosystem. This contamination on these lands in different crops causes a decrement in growth, performance, and yield [38].

Heavy metals affect humans and other living organisms through contaminated food and water, food chain, polluted soil, reduction in food quality, and reduction of fertile and culturable lands for farming [39–41]. Several HMs are essential in low concentration for living organisms (Mn, Cu, Zn, Mo, and Ni), but these elements are very toxic in high concentration [37].

### Lead

Lead (Pb) is one of the most common HM contaminants in the soil. This metal is highly toxic to living organisms and can cause biochemical, morphological, and physiological dysfunctions in plants and other living organisms [42]. In metalloproteins, Pb enters the cells with the help of Fe^2+^ and Ca^2+^ transporters and creates toxicity by displacing Fe^2+^ and Ca^2+^ cations at their binding sites [23]. Lead is a pollutant that accumulates in soils, water, and sediments and is stable in the environment. Anthropogenic activities such as mining, fuel combustion, smelting, synthetic fertilizers, and various industrial processes (e.g., building construction, bullets and shot, solder, pewter, Pb-acid batteries, fusible alloys) have increased in recent decades and these activities influence the global cycles of Pb [42, 43]. Among the natural factors, it can be pointed weathering and parent rock erosion, and volcanic eruptions that transfer Pb to water bodies and lands [44, 45]. This metal is not biodegradable and is greatly stable in water and soil [42, 46]. Lead can enter the plant cells through this pathway: At high concentrations, physical barrier in the plant is broken, and Pb enters the plasma membrane with the help of ion transporters. Then PCs (phytochelatins) chelate Pb, and this complex is sequestered in the vacuole compartments. Also, Pb can be transported to the aerial parts through the drainage vessel [35]. According to the US Centres for Disease Control and Prevention and the World Health Organization, a blood lead level of 10 μg/dL or above is a cause for concern [47].

### Copper

Copper (Cu) is an essential and low-consumption element. This metal plays a role as an enzymatic cofactor (respiration and electron transport proteins) in living beings. Copper has toxic effects on cells at high concentrations (3 mg/L and greater) due to the disruption of cell membrane integrity, its interaction with nucleic acids, interference with the energy transport, and disruption of enzyme activity [23, 48–50]. In the cytoplasm, copper competes with other metals for conjoining to the protein binding sites that can lead to dysfunctioned proteins. Also, in combination with hydrogen peroxide (H_2_O_2_), this element produces hydroxyl radicals that have harmful effects on DNA, lipids, and other molecules [23, 51, 52].

### Cadmium

Cadmium (Cd) enters the environment in high concentrations through urban residues, mining, smelting, metal manufacturing, and synthetic phosphate fertilizers [53]. When the total cadmium (Cd) concentration in soil exceeds 8 mg kg^−1^ (bioavailable Cd concentration becomes > 0.001 mg kg^−1^) and in plant tissue reaches 3–30 mgkg^−1^, most plants exhibit visible Cd toxicity symptoms [54]. This metal has biological activity in all living organisms [55].

The effects of cadmium toxicity in plants can be mentioned as follows: decreases the uptake and displacement of nutrients and water, increases oxidative damage, interrupts plant metabolism, morphology, and physiology processes [53].

Also, the effects of cadmium toxicity in humans can be mentioned as follows: the effect on kidneys (renal tubular damage, pulmonary emphysema, and kidney stones) [56]; replaced with calcium (Ca) in human body, and reduce Ca’s uptake [57, 58].

### Chromium

This metal exists in three different oxidation forms, including Cr(0), Cr(III), and Cr(VI) species. The toxicity of chromium (Cr) depends on its mobility in plants so that Cr(VI) is toxic because of its high mobility. The median lethal dose of hexavalent chromium is 50–150 mgkg^−1^ [59]. At the same time, Cr(III) is less mobile and less toxic. Plants take up Cr by carriers like sulfates, and Cr toxicity in plants shows signs like a decrease in seed germination, a reduction of growth and yield, prohibition of enzymatic activities, photosynthesis disturbance, mutagenesis nutrient, and oxidative imbalances [60]. This metal is toxic to plants, aquatic animals, and microorganisms and is considered a human carcinogen [60–62]. High chromium accumulation in edible plants can represent a potential danger to animals and humans [63, 64].

### Mercury

Mercury (Hg) is a toxic, stable, and mobile contaminant; this metal is very volatile in nature and can be transported within air masses at very long distances [65]. The worrisome level of soil contamination in farmland (4 mgkg^−1^) and industrial areas or factories (16 mgkg^−1^) is suggested [66]. Mercury causes pollution in the environment through the following cases: (1) petrochemicals, (2) painting, (3) mining, (4) agricultural inputs (fertilizer, fungicidal, and sprays), (5) household bleach, (6) chemicals (e.g., battery acid), (7) household lye, (8) muriatic acid (hydrochloric acid), (9) sodium hydroxide, (10) medical instruments, (11) thermometers, (12) barometers, (13) manometers, (14) fluorescent lamps, (15) batteries, (16) incandescent wire filaments, (17) mercury vapor lamps, (18) ultraviolet lamp, (19) pesticides, (20) laboratory chemicals, (21) inks and paper coatings, (22) wiring devices and switches, (23) lubrication oils, (24) textiles [67]. Mercury interferes with the transportation of electrons in chloroplasts and mitochondria through photosynthesis and oxidative metabolism. Plants do not take in as much water when this metal is present. It also affects the brain by poisoning it and causes neurological and renal problems [68, 69].

### Zinc

Zinc (Zn) is one of the essential trace elements for all living beings. Zinc in low concentrations is a cofactor for many proteins and have a structural and functional role [70, 71]*.* Nevertheless, this element is very toxic at high concentrations, and when plants highly absorb this element it can be harmful to consumers [71, 72]. Minimum thresholds for adverse effects of dissolved zinc on aquatic organisms are 50–100 µg^−l^, acute effects on mysids; 100–200 µg^−l^, acute effects on fish; 200–1000 µg^−l^, acute effects on amphipods and decapods; and 1000–10,000 µg^−l^, acute effects on polychaetes and mollusks [73].

### Arsenic

Contamination of the human’s food chain with arsenic (As) is a worldwide concern that causes irreparable damage to the health of living organisms, especially humans [74]. Arsine gas is the most toxic form of arsenic inhalation of over 10 ppm which is lethal [75]. This HM is not essential and generally is toxic to plants. Absorbed As by plant roots prevents expansion and proliferation of root. Arsenic strongly prevents plant growth by attenuating expansion and biomass accumulation, fertility losses, and yield and fruit production [76]. Arsenic has two forms, including arsenate (AsV) and arsenate (AsIII) that these forms could be taken up by the cells of the plant roots. These forms (AsV and AsIII), through distinct mechanisms, disrupt plant metabolism. AsV is a chemical analog of phosphate that, to some extent, can disrupt certain phosphate-dependent aspects of metabolism by phosphate transport proteins. Also, this form during unstable reactions and phosphorylation of AsV causes short-lived [74]. Therefore, exposure of plants to As can be resulted in many morphological, physiological, and biochemical changes [75]. Arsenic toxicity depends on its form. Generally, the pH, surrounding mineral composition, redox conditions, and microbial activities affect this metal’s inorganic or organic form and its oxidation state [67, 77].

### Nickel

In the plant growth period, nickel (Ni) is an essential micronutrient and a component of the urease enzyme for nitrogen metabolism in plants. Chlorophyll content in maize with increased concentration of Ni from 20 to 100 μM and the fresh weight of shoots of sunflower decreased with increasing concentration of Ni from 10 to 40 mgL^−1^ [78]. This metal in plants can be very toxic at high concentrations. Activity of antioxidant enzymes such as superoxide dismutase (SOD), ascorbate peroxidase (APX), and catalase (CAT) can be changed by Ni. Also, this metal inhibits growth, induces chlorosis, necrosis, and wilting, and in non-tolerant plants, elevated levels can inhibit root meristem cell division and reduce plant growth. Generally, this metal can inhibit protein and chlorophyll synthesis, decrease water content at high concentrations, and exert a negative effect on photosynthesis [79].

### Bioremediation

Anthropogenic activities and industrialization of countries have created environmental contamination that has had irreversible effects on the lives of all living organisms and microorganisms. With the development of industry and increasing HM concentration in the soil, we must look for a way to reduce the amount of these pollutants or neutralize the toxic effects of these metals. Therefore, finding a way to limit the harmful effect of these pollutions is vital [80]. Physico-chemical and biological methods can be applied to neutralize HMs’ toxic effects on the environment [81]. Living organisms can convert toxic pollutants into less toxic forms reducing, through detoxifying, degrading, and mineralizing mechanisms [80]. Bioremediation is done in order to eliminate pollutants from the biosphere for living organisms. The biological processes (such as degrade, detoxify, and even accumulate harmful organic and inorganic compounds) carried out by these organisms reduce the environmental effects of these pollutants [82].

### Microorganisms Used in Bioremediation

Due to long-term toxic effects, HM pollution has critical issues for all life forms in the environment. Because of the durability of pollutants and organic metals, these substances persist for a long time in the environment and can negatively affect the food chains of organisms even in low concentrations. Using physical and chemical methods to remediate soil of contaminated areas is not economical and produces a lot of chemical wastes [83]. Microorganisms used in bioremediation are indigenous or non-indigenous, which they can be introduced to contaminated sites in different ways. Using indigenous microorganisms in contaminated environments is the most important approach that challenges solving problems related to biodegradation and bioremediation of pollutants [84]. These microorganisms are eco-friendly and cost-effective and have various mechanisms for metal sequestration and metal biosorption. Adaptability and biologically activated systems in bacteria make them suitable for the remediation process [85].

Several bacterial species have been tested for bioremediation, such as *Flavobacterium*, *Achromobacter*, *Alcaligenes*, *Corynebacterium*, *Flavobacterium*, *Mycobacterium*, *Nitrosomonas*, *Xanthobacter, Pseudomonas*, *Bacillus*, *Enterobacter*, and *Micrococcus* sp. Their remarkable biosorption ability is because of their high surface-to-volume ratio and potential active chemisorption sites (teichoic acid) on their cell wall [14, 86]. It has been reported that when the population of indigenous microorganisms capable of degrading the target contaminant is less than 10^5^ CFU/g of soil, bioremediation will not occur at a significant rate [87]. The carrier material used for the carrier-based inocula is corncob powder (very good soil conditioner). This carrier material has augmented the degradation rate by providing air pockets in the soil, thereby making it porous and facilitating aeration for growth and survival of the introduced bacterial consortium and bioremediation. The type of substrate (the treatment consisting of the selected bacterial consortium and nutrients) and the concentration of inoculum lead in the maximum bioremediation response [88]. An experiment was performed to evaluate the saprophytic survival of *Phytophthora drechsleri*. Sampling was carried out on soils around pistachio trees in various regions of Rafsanjan, Iran. The results showed that the type of substrate (wheat straw and pistachio leaf), incubation time, and inoculation density play an important role in fungal survival, so that increasing inoculum density would result in longer survivability of *P*. *drechsleri* [89].

### The Bioremediation Types

Bioremediation can be done in different ways including detoxification, degradation, mineralization, or transformation of toxic pollutants to a less toxic form. The method we use in bioremediation depends on multiple factors such as cost, site characteristics, type, and concentration of pollutants, and can be performed either ex situ or in situ [80, 90].

### In Situ* Bioremediation*

In situ bioremediation involves treating polluted substances at the site of contamination. This technique does not require any excavation; it means that we have little or no soil disturbance. Since there is no need to dig the ground, this technique is low-cost [91]. This technique has effectively treated HMs, chlorinated solvents, dyes, and hydrocarbons in polluted sites [91, 92]. Intrinsic and engineered bioremediations are two different techniques used in in situ technology. Intrinsic bioremediation involves the remediation of polluted soils without any external force. So that deals with incitement of indigenous or naturally occurring microbial population and is less expensive. Engineered bioremediation uses engineered microorganisms and improved physicochemical conditions in order to increase the degradation rate of pollutants [80].

### Ex Situ* Bioremediation*

**Fig. 3:**
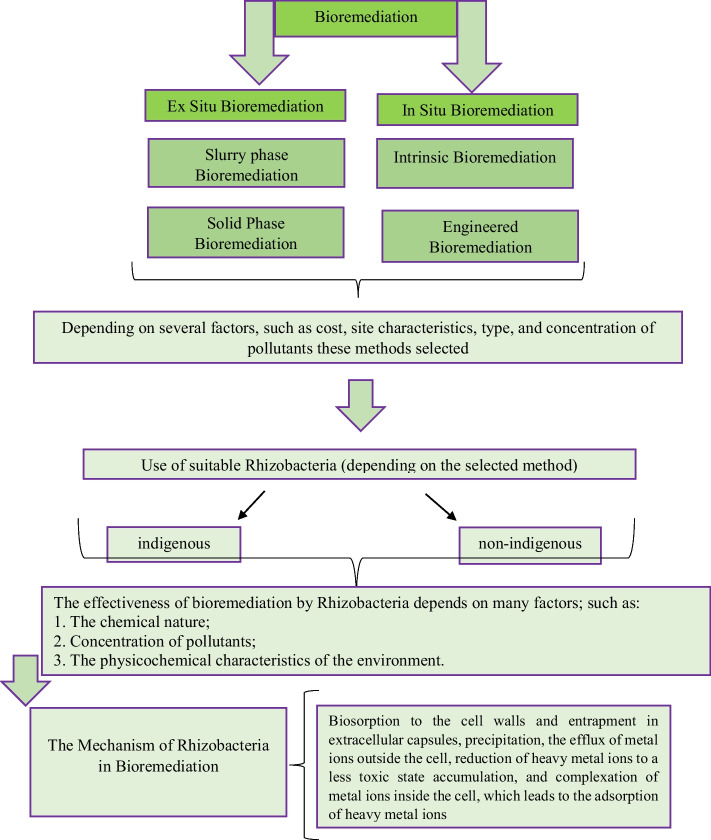
Bioremediation approaches, the use of rhizobacteria, and their mechanisms

Ex situ bioremediation involves digging pollutants and displacing them from contaminated sites to another site for treatment. In ex situ, bioremediation techniques have been done based on the depth of pollution, pollutant type, pollution degree, treatment cost, and geographical location of the contaminated site. In this method, pollutants are dug and then transported from polluted sites to another site for treatment [80]. Solid-phase treatment and slurry-phase bioremediation are two forms of ex situ technology. In solid-phase bioremediation, after soil excavation, bacterial inoculum is released into soil piles with the help of pipes in order to remediate HMs [93]. In slurry-phase bioremediation, polluted soils are combined with water, nutrient, and oxygen in a bioreactor to provide an optimized situation for the microorganisms to efficiently degrade the soil contaminants [80]. Figure 3 shows the types of bioremediation, the use of rhizobacteria, and their mechanisms.

### Bioremediation with Rhizobacteria

The industrialization of countries is increasing so that environmental pollutants enter the environment quickly. These pollutants are harmful for living organisms, so we should choose natural methods that positively affect living organisms to control pollutants. Regeneration of polluted sites using bioremediation is eco-friendly and (microbial process and beneficial microorganisms) has been proven effective [80].

Using PGPRs for bioremediation is a natural technique that can be used to reduce the adverse effects of environmental pollutants.

Guo et al. [94] collected *S. nigrum* (a cadmium hyperaccumulator) from a sewage discharge canal bank, after surface sterilizing isolated endophytic bacterial strains. Bacterial strains were maintained and activated in LB medium and evaluated for their ability to limit the effects of HMs (Cd(II), Pb(II), Cu(II), Cr(VI), and Zn(II)) in soils. The selected strains were identified by the determination of 16S rDNA gene sequences. In this research, 96 were isolated strains, and the EB L14 strain belonged to *Bacillus* spp. This strain’s morphological, physiological, and chemical characteristics had an excellent performance to remove Cd(II) and Pb(II). At the 100 mgL^−1^ concentration of cadmium ions, the lag phase was expanded, and the maximal cell density was reduced below 23.86% of the control. This shows that the lag phase and optical density of endophytic bacteria depend on HMs’ concentration and toxicity.

In HM hyper accumulator plants, endophytes confront challenges in many ways such as greater demand for energy to stand with the pollutant’s toxicity. Subsequently, the continuing usage of energy (ATP) enforces the cells into some altered growth rhythm, which, in a long time, causes enhanced growth of EB L14. In four mediums, the relative growth curves of EB L14 showed that only the divalent cations of HMs (such as C(II)) could induce the ATPase to generate more ATP for the endophyte to overcome the toxicity of these divalent HMs [94]. Generally, the result of this research showed that strain EB L14 (*Bacillus* spp.) has a multi-metal resistance (Cd, Pb, and Cu) that is because of the prohibition of the ATPase activity [94].

**Fig. 4:**
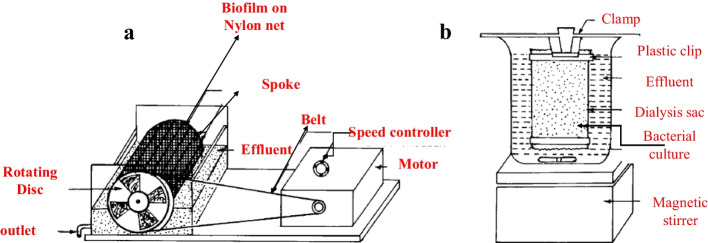
Rotating biological contactor (**a**) and dialysis bioreactor (**b**)

Ganguli and Tripathi in 2002 [95] tested the chromate-reducing ability of *Pseudomonas aeruginosa* with three methods, including batch culture, cells entrapped in a dialysis sac, and cells immobilized in an agarose-alginate film in conjunction with a rotating biological contactor. A2Chr strain was selected in this experiment. This strain was isolated from the effluent of a leather-tanning unit. In this research, a laboratory-scale rotating biological contactor of standard design [96] was fabricated (Fig. 4a). Figure 4b shows dialysis tubing in sterile triple-distilled water.

The result of this research showed that the rotating biological contractor could be a very efficient approach to bioremediate chromium from industrial effluents, but some methods should be extended for making stable biofilms consist of chromate-reducing strains. Free cells can also be made to reduce higher amounts of Cr(VI) provided that they are respiked with 10 mg Cr(VI)/l at regular intervals. Since *P. aeruginosa* A2Chr just only reduces Cr(VI) in an effluent containing C, N, and P, the effluent should also be supplemented with appropriate sources of C, N, and P. Detoxification of chromium from industrial effluents can be done economically if chromium is mixed with nutrient-rich wastewater (e.g., sewage), in order to provide essential energy and nutrients for bacteria to decrease Cr(VI) in the mixed effluent [95].

In other research, bacteria which are resistant to HMs were isolated from different industrial locations in India. These bacteria were tested for metal resistance against CdP2 + P, NiP2 + P, HgP2 + P, CuP + 2P, and PbP2 + P by determining the minimal inhibitory concentration ranging from 10 to 250 μg/ml [97]. One gram of fresh soil was dissolved 1 ml in sterile distilled water, and 0.1 ml from each of these dilutions was placed on the culture medium containing 10 g peptone, 5 g yeast extract, 10 g NaCl, and 15 g agar, with pH 7.2. Also, 10 μg/ml cadmium nitrate was added to this medium. Then the culture medium was incubated (24–48 h, 30C), and colonies that were different in shape were selected and purified. Bacterial growth was assessed by measuring optical density (OD) at 620 nm. The concentration of the metal which allowed bacteria to grow and the concentration which no growth was observed was considered the minimum inhibitory concentration of the metal against tested strain. Producing hydrogen sulfide is one of the probable mechanism that provides resistance to cadmium. In this experiment, alkaline lysis method was used to screen the presence of plasmid DNA in bacterial isolates [97, 98]. Other parameters measured include (1) production of sulfide, (2) determination of cadmium sulfide precipitation, (3) accumulation of cadmium, and (4) removal assay in the presence of thiosulfate [97]. These strains (including both gram-positive and gram-negative (MF1, MF2, MF3, MF4, and MF5)) were able to grow and tolerate an elevated level of metal toxicity. All bacterial isolates showed the presence of megaplasmid except MF1. The result of plasmid curing for identifying whether the HM resistance gene was encoded by plasmid or chromosome showed that after plasmid curing, bacterial growth was retarded on a medium amended with Cd, but it was not prohibited completely. Bacteria use a different mechanism to neutralize the toxic effects of HMs. These mechanisms include (1) ATPase-dependent efflux mechanisms; (2) precipitation of HMs (Cd2 +) on the cell surface of bacteria [99]; (3) binding of cadmium to bacterial capsules (in *Arthrobacter viscosus* and *Klebsiella aerogenes*); (4) HMs can enter the cell as an alternative substrate for cellular ions transport system [97, 100–102]. Bacterial plasmids (for example, CadA and CadB operons) contain genes that allow bacteria to be resistant to toxic HMs, including Cd, Bi, Cu, Hg, Cr, and Zn. These plasmids encode genes which are responsible for resistance to antibiotics and HMs [97, 103]. In some strains (MF1 and MF2), detoxification is achieved by entrapment of HMs in extracellular polymers. In bacteria, extracellular polymeric substances play an essential role in metal sorption and establish a passive method. The metal cations bind to the negative charges of acidic groups from exopolysaccharides [97]. The production of hydrogen sulfide in some isolates (MF3, MF4, and MF5) removes cadmium in the form of cadmium sulfide (insoluble CdS can detoxify cadmium) [97]. In industrial areas, heavy metals are one of the most important abiotic factors that can endanger plant health, but beneficial microorganisms by effective mechanisms can alleviate this stressful condition. In a study, wild-type *Pseudomonas aeruginosa* (named Pse-AB) which was tolerant to Cd was isolated from the fields contaminated with oil in China and was considered a control strain [104]. In this strain under in vitro conditions, the ability of phosphate solubilization, producing siderophores, indole acetic acid (IAA), and hydrogen cyanide was assessed in both the presence and the absence of Cd^2+^ [104]. In this study, green peas (*Pisum sativum* L.) were selected for greenhouse experiments. The strain of Pse-w that is isolated from HM-contaminated soils is a gram-negative bacterium. This indigenous strain could tolerate up to 4 mM CdCl_2_ and is more tolerant to Cd than Pse-AB (non-indigenous strain). All plant growth–promoting activities (production of salicylic acid, IAA and phosphate solubilizing activity, siderophore activities, 1-aminocyclopropane-1-carboxylate (ACC) deaminase, and solubilization of inorganic phosphorus) in the strain Pse-w were more significant than those in Pse-AB. Also, using Pse-w in contaminated soil enhanced root and shoot growth, and chlorophyll content in green peas also caused 83.33% seed germination [104]. These strains, by producing siderophores and IAA, enhance mineral and nutrient uptake to improve lateral and adventitious root growth and prevent plants from becoming chlorotic [104–106]. An interesting result was obtained in this study: western blot showed that a hybrid fusion protein inaKN/MT efficiently expressed and provided a threefold increase in their metal-coordinating capacity and conferred more protective effect upon *Pisum sativum* L. growth in polluted soils [104]. Metallothioneins (MTs), small cysteine-rich metal-binding proteins, support the viability of organisms under different environmental stressors especially HMs [107]. Cysteine-rich MTs are used for bioabsorption of metal ions and increase the Cd immobilization by bacterial cells which are the main metal-sequestering molecules [104, 108, 109]. In a research, six rhizobacterial strains (*Pseudarthrobacter*, *Pseudomonas*, *Rhodococcus*, and *Stenotrophomonas*) isolated from highly heavy metal–contaminated soils situated in mining areas were tested with a native leguminous plant in Oujda region. These strains were multi-resistant to heavy metals (chromium, copper, lead, zinc, and arsenic). Inoculation with the *Rhodococcus qingshengii* strain LMR340 boosted plant biomass compared to uninoculated plants, chlorophyll and carotenoid content, and antioxidant enzyme activities [110].

Copper is one of the heavy elements whose effect on plants was mentioned previously.

There are three resistance systems in microorganisms in interaction with copper [23]:

1. PCO (the periplasmic plasmid-borne copper resistance system) encodes a multi-copper oxidase protein responsible for the oxidation of Cu(I) in the periplasmic space. This system has a high resistance performance against copper [111–113].
2. Microorganisms have an ATPase pump that can lead copper ions outside [52, 114].
3. Cation sensing copper efflux system (Cus system), which belongs to the resistance nodulation-cell division family encodes CusA protein which is responsible for HM exportation [52, 113–116].

Copper sulfate is applied in vineyards for controlling fungal diseases, so copper rapidly absorbs into the soil of these areas and causes environmental pollution. Bacteria which absorb copper efficiently can be applied for bioremoval of copper from polluted sites [117]. Andreazza et al. [117] were screened strains for copper bioremoval, by DNA-based methods to reconnoiter promising copper-resistant isolates with the potential ability to remove copper from contaminated environments. Isolates were identified by 16S ribosomal RNA gene sequencing. Most of the isolates were identified as *Bacillus* species [117]. Also, other research showed that *Bacillus* is a substantial bacterial genus to bioremediate HMs in different contaminated areas [118, 119]. This property may be due to the cell wall components of prokaryotes, which include a set of functional groups with metal binding capacity [120]. Table 1 lists other studies on bioremediation of heavy elements.

**Table 1:** The list of rhizobacteria and their mechanisms in the bioremediation of heavy metals

Rhizobacteria	Heavy metal	Result	Reference
*Bacillus megaterium*	Pb	Intracellular cytoplasmic leads to accumulation	[121]
*Ralstonia metallidurans*	Pb	Three-component divalent-cation efflux systems (chemiosmotic pumps)	[122]
*Pseudomonas marginalis*	Pb	Extracellular leads to exclusion	[121]
*Staphylococcus aureus* and *Citrobacter freundii*	Pb	Intracellular lead-phosphate	[123]
*Pseudomonas putida*	Zn, Cd	P-type ATPases and two CBA transporters	[115]
*Frankia* sp.	Pb	Cells’ accumulated Pb^2+^ with saturation kinetics, indicate different Pb–PO_4_ compounds formed	[124]
*Streptococcus thermophilus*	Cd, Zn	two genes (cadCSt and cadASt) to constitute in cadmium/zinc resistance	[125]
*Xanthomonas citri* subsp. *citri* and *X. alfalfae* subsp. *citrumelonis*	Cu	Open reading frames (ORFs) related to the genes copL, copA, copB, copM, copG, copC, copD, and copF to be present on a large (~ 300 kb) conjugative plasmid	[48, 126]
*Frankia*	Cu	Copper was accumulating inside of Frankia or binding to the cell surface	[127]
*Acidithiobacillus ferrooxidans*	Cu	By formatting phosphate granules through stimulation of polyphosphate hydrolysis and formation of copper-phosphate complexes	[128]
*Burkholderia fungorum*	Zn, Pb, and Cd	Metal accumulation in the cell wall and intracellular space of strain. Catabolic activity and the ability to tolerate high concentrations of toxic metals	[129]
*Burkholderia dabaoshanensis* sp. *nov*	Cd	The adsorptive mechanism for cadmium (action of the amide, carboxy, and phosphate of cell surface and producing low-molecular-weight (LMW) organic acids to complex or chelated Cd^2+^)	[130]
*Burkholderia* sp.	Zn, Pb, Mn, Cd, and Cu	The adhesion of heavy metal–contaminated soil minerals with *Burkholderia* sp. and the formation of a metal complex with biosurfactant	[131]
*Enterobacter cloacae*	Cr, Cd, Ni, and Pb	The order of toxicity of the heavy metals were Cd > Cr > Pb > Ni. With increasing concentrations of heavy metals, there was a reduction in P solubilization, as well as a decrease in the pH of the liquid medium and inhibition in the development of bacterial biofilms	[132]
*Klebsiella variicola*	Pb, Cd, and As	Artificially mutated strains of *K. variicola* may be applied to remove cadmium from a polluted environment	[133]
*Alcaligenes faecalis*, *Bacillus pumilus*, *Pseudomonas aeruginosa*, and *Brevibacterium iodinium*	Cd, Pb	Detoxify hexavalent chromium, remove 70% and 75% cadmium (Cd) with a reduction of 1000 mg/L to 17.4 mg/L of cadmium (Cd) by *P. aeruginosa*, and to 19.2 mg/L by *A. faecalis* in about 72 h	[24]
*Bacillus mycoides* and *Micrococcus roseus*	Cd	Increased biomass and shoot nutrient uptake of maize by bacterial treatments in compared with control in the soil polluted with Cd	[134]

## Conclusion

**Fig. 5:**
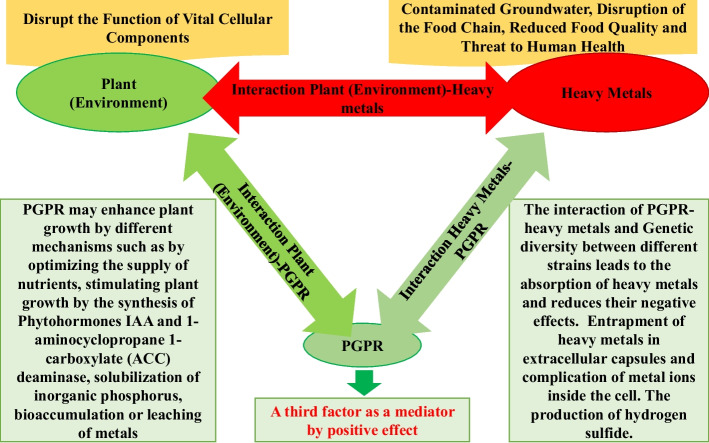
Interaction between plant (environment)-heavy metal-PGPR

Heavy metal accumulation in soils greatly impacts the performance, morphology, and physiology of plants and will also affect human life [135, 136]. HMs will eventually affect the plant tolerance threshold and cause changes in the plant genome and genetic structure. Changes in the plant genome lead to changes in encoding proteins. Human and animal usage of these mutated plants causes many diseases in humans and animals. The interaction between the plant (environment) and HMs leads to negative interactions that affect plant activities. HMs as an inanimate and at the same time, harmful factors have irreversible effects on the environment. Therefore, there is a need for a third factor as a mediator to neutralize the negative interaction between the plant and the heavy elements. PGPRs can act as a mediator to address the adverse effects of this interaction. Figure 5 shows the interactions between plant-HMs and the presence of PGPR as a mediating factor. Furthermore, methods of isolating metal-resistant bacteria from the polluted sites for bioremediation offer engaging perspectives. In these bacteria, the existence of genetic diversity and adaptation to the geographical and climatic conditions of contaminated areas help them reduce the negative impact of heavy elements in the environment through various mechanisms.

## Data Availability

Not applicable.
